# Sevoflurane-mediated modulation of oxidative myocardial injury

**DOI:** 10.34172/jcvtr.2023.31724

**Published:** 2023-09-23

**Authors:** Siavash Sedghi, Wiam Z. Khadra, Leili Pourafkari, Paul R. Knight, Faraz A. Alderson, Nader D. Nader

**Affiliations:** ^1^Department of Anesthesiology, University at Buffalo, Jacobs School of Medicine and Biomedical Sciences, Buffalo, New York, USA; ^2^VA Western New York Healthcare System, Buffalo, New York, USA; ^3^Cardiac Imaging, The Lundquist Institute, Harbor-University of California at Los Angles Medical Center, Los Angles, USA; ^4^Faculty of Engineering, University of Guelph, Ontario, Canada

**Keywords:** Apoptosis, Necrosis, Reactive oxygen species, Volatile anesthetics

## Abstract

**Introduction:**

Volatile anesthetics offer protection when administered throughout an ischemic injury. We examined how volatile anesthetics modulate the cardiac myocytic injury associated with hydrogen peroxide.

**Methods:**

Forty-eight Long-Evans rats were divided into four groups depending on the treatment: none (CONT), Glibenclamide (GLB); Sevoflurane (SEV); or GLB+SEV. Each group was further divided into two, one of which was exposed to hydrogen peroxide (H_2_O_2_). Oral GLB was administered 48 hours before myocardial isolation. All rats were anesthetized by intraperitoneal injection of Ketamine, and the hearts were harvested after heparinization. Cardiomyocytes were isolated using a combination of mechanical mincing and enzymatic digestion. After isolation, the aliquots of cells were exposed to H_2_O_2_ and FeSO_4_ for 30 minutes. The cell suspensions were then bubbled for 10 minutes with 100% oxygen and 1.5% SEV if appropriate. Apoptosis was detected by fluorescein-bound annexin-V (ANX-V), necrosis by propidium iodide, and ELISA assessed caspase-3 activity in all groups.

**Results:**

There was an increase in apoptosis, necrosis, and caspase-3 activity in the cells following exposure to hydrogen peroxide. SEV reduced the rate of cell necrosis and apoptosis. Pretreatment with GLB did not alter the effects of SEV. Similarly, caspase-3 activity did not change with GLB, although SEV administration reduced this enzymatic activity in response to hydrogen peroxide.

**Conclusion:**

In this oxidant injury model, we demonstrated that incubating isolated cardiomyocytes with SEV profoundly diminished H_2_O_2_-induced apoptotic and necrotic cells compared to their CONTs. These results support the hypothesis that K_ATP_ channels are not the sole mediators associated with anesthetic preconditioning.

## Introduction

 Reactive oxygen species (ROS) are integral to many inflammatory and noninflammatory diseases. Although they also play a role in the normal homeostasis of the cell, an overabundance of ROS leads to a robust inflammatory response which directly or indirectly leads to cell death. Inflammatory responses associated with ROS have been well-studied in diseases such as cancer, rheumatic and neurodegenerative diseases, diabetes mellitus, and ischemia-reperfusion injuries.^1,2^ The importance of the injurious effects of ROS can be demonstrated in ischemic organs, such as the heart, following an acute myocardial infarction. For example, during myocardial infarction, the central nidus of the ischemic myocardium becomes necrotic and will later heal through scar formation. The myocardium adjacent to the infarct (the penumbra zone) following ischemia is functionally impaired (stunned) even though the myocardium may still be viable. Depending on the extent of ROS present, this penumbra zone may return to its normal function or become apoptotic, ultimately leading to heart failure.

 It has been well-established that volatile anesthetics are potent immune modulators. They have decreased inflammatory responses, leading to cell death by either apoptosis or necrosis. The exact mechanism of action has yet to be well understood, but ATP-gated potassium (K_ATP_) channels have been primarily implicated. Our team has previously demonstrated decreased inflammatory-related injury associated with ischemia-reperfusion injury in cardiopulmonary bypass patients.^3^

 Our objective is to examine how volatile anesthetics modulate ROS-associated cellular injury. Based on our previous experimental and clinical findings, we hypothesize that volatile anesthetics have protective effects against apoptosis and necrosis in cardiomyocytes. We further hypothesize that this protection is primarily mediated through the activation and opening of K_ATP_ channels. To test this hypothesis, we will examine apoptosis and necrosis in isolated cardiomyocytes with inactivated K_ATP_ channels.

## Materials and Methods

###  Experimental design

 Forty-eight healthy male Long-Evans rats (Harlan-Sprague Dawley, Indianapolis, IN) with body weights of 250-300g were randomly divided into four different treatment groups: no treatment (CONT); Glibenclamide (GLB); Sevoflurane (SEV); or GLB with SEV. Each of these four treatment groups was randomly divided into two, one exposed to hydrogen peroxide (H_2_O_2_) (Table 1). Forty-eight hours before the experiment, all treatment groups involving GLB (groups 3, 4, 7, and 8) were treated once with 10mg/kg of GLB (Sigma Chemical Company, St. Louis, MO, United States) to induce deactivation of K_ATP _channels. The treatment was administered through an 18-gauge gavage feeding tube about three inches long to ensure proper administration.

**Table 1:** Treatment and intervention groups

**Number**	**Group Name**	**Number of animals**
Group 1	No insult Controls (Negative Controls)	6
Group 2	Oxidative insult Controls **(Positive Controls)**	6
Group 3	Glibenclamide without Oxidative insult (Treatment Controls)	6
Group 4	Glibenclamide** with** Oxidative insult	6
Group 5	Sevoflurane without insult	6
Group 6	Sevoflurane **with** Oxidative insult	6
Group 7	Glibenclamide + Sevoflurane without insult	6
Group 8	Glibenclamide + Sevoflurane **with **Oxidative insult	6

###  Anesthesia and myocardial tissue harvest cardiomyocyte isolation (Enzymatic Digestion)

 The non-SEV groups (1-4) were anesthetized using ketamine (25 mg/kg) intraperitoneally (IP). The remainder of the groups (5-8) were anesthetized with SEV until animals had no pedal reflex after a firm toe pinch. SEV vapor (2%) with oxygen was delivered through a nose cone to maintain the anesthesia in groups 5 through 8. For tissue harvest, animals were heparinized (10,000 U/kg) (JA Webster, MA) with an injection into the ear vein to prevent clotting in the coronary arteries. Medial sternotomy was made by exposing the heart and ascending aorta by dissection of the pericardium. The heart was lifted anteriorly, exposing the pulmonary artery and the ascending aorta. The heart was isolated by transecting the proximal ascending aorta, leaving enough arterial stalk attached (proximal to the inanimate artery) to allow cannula placement without the aortic root. The heart was then removed and placed in a cold cardioplegic solution (modified Krebs-Hensleit solution) and was massaged to remove access blood from the chambers and the vasculature system before weighing by dripping a Krebs–Hensleit (KH) solution through the aorta.

 The cells were then enzymatically broken up by breaking the calcium (Ca^2+^) bonds, subsequently digesting collagen and other connective tissues (KH solution without and with CaCl_2_ / collagenase, respectively). The heart was then mechanically diced into small pieces and incubated with KH solution with trypsin to break extracellular proteins and cleavage of gap junctions without disturbing the cells’ integrity. Trypsin activity was then stopped by adding Ca^2+^ back into the system. After a manual cell count, 250 000 cells were washed and resuspended in 5 milliliters of sterile KH solution.

###  Post-Isolation Treatment and Oxidative Injury

 The CONT treatment groups (1 and 2) were exposed to 100% O_2_ for 30 minutes after isolation of the individual left ventricular myocytes. This was done by bubbling oxygen gas into the KH solution containing said cells at a flow rate of 400 mL/minute. Similarly, SEV-treated groups (5-8) were exposed to SEV at 2% and oxygen at 98% for 30 minutes using the same method. All cells were incubated in KH solution (pH = 7.4) at 37°C with 95% humidity and aerated with carbogen (O2 95% + CO_2_ 5%).

 Fenton reaction was utilized to induce oxidative damage to the cardiomyocytes by adding H_2_O_2_ and FeSO_4_ (groups 2, 4, 6, and 8). Cell suspensions were treated with 100μM H_2_O_2 _(Sigma Chemical Company, St. Louis MO, United States) and 200μM anhydrous FeSO_4_ (Sigma Chemical Company, St. Louis MO, United States). To stop the Fenton reaction, a mixture of 2 millimolar concentration (mM) of sodium etidronate (Sigma Chemical Company, St. Louis MO, United States) and 1mM sodium thiosulfate (Sigma Chemical Company, St. Louis MO, United States) in phosphate-buffered saline (PBS) (Burlington, MA, United States) were then added to the cell solutions. The cells were then subsequently washed with PBS.

###  Determination of Apoptosis / Necrosis

**Figure 1:**
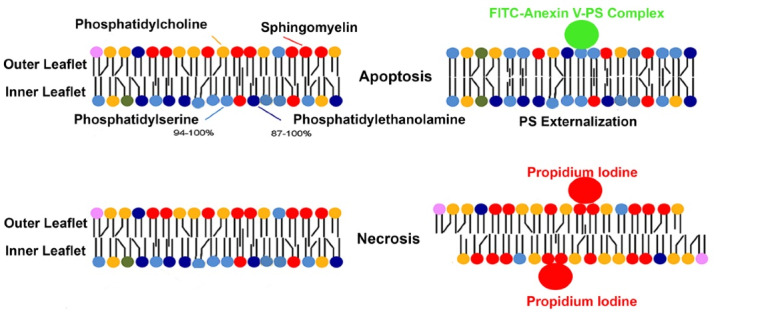
The mechanism of fluorescence of Annexin-fluorescein isothiocyanate (FITC) and Propidium iodide (PI)

 Annexin-5 (ANX-V) is a 35-36 kDa Ca^2+^ -dependent phospholipid-binding protein with a high affinity for phosphatidylserine (PS). Cells undergoing apoptosis have PS, usually on the inner leaflet translocated to the outer layer in the early apoptosis.^4,5^ ANX-V may be conjugated to a fluorochrome, including fluorescein isothiocyanate, thus detected by flow cytometry. Propidium iodide (PI) is used in conjunction with ANX-V on cells with a cell permeability (i.e., cells that have undergone necrosis).^4^ The nucleic acid intercalation bound to PI can also be visualized under flow cytometry (Figure 1).

 A commercially available fluorescein isothiocyanate ANX-V Apoptosis detection Kit (Pharminogen, United States) was used to distinguish between apoptotic and necrotic cells. Briefly, the cells were washed twice with cold PBS and resuspended. 5 µl of fluorescein isothiocyanate - ANX-V and 5 µl PI were added to 100 000 cells. The cells were then gently vortexed and incubated for 15 minutes at room temperature (25°C) in the dark, and a binding buffer was added and analyzed using flow cytometry.

###  Caspase 3 assay

 Caspases are crucial mediators of apoptosis, among which, Caspase 3 is an activated protease.^6-8^ To test for caspase 3 (a common mediator of the apoptotic pathway), we used a commercially available Caspase-3 Assay Kit (Abcam, Cambridge, UK). Briefly isolated left ventricular myocytes from all groups were lysed and incubated with DEVD-AFC (substrate for activated caspase-3) for 2 hours. The fluorescence intensities of the treated samples were compared using a Bio-Rad plate reader.

###  Data Management and Statistical Analysis

 Six rats were studied in each group. The number of rats in each group was selected based on power analysis accepting a 20% beta error and 5% alpha error. Data were expressed as mean ± SEM and analyzed using a one-way analysis of variance with the Bonferroni *post hoc* test. Student’s t-tests were used for intergroup comparisons. Bonferroni correction was utilized for multiple comparisons and intergroup analyses. Null hypotheses were rejected at *P* value < 0.05.

## Results

**Figure 2:**
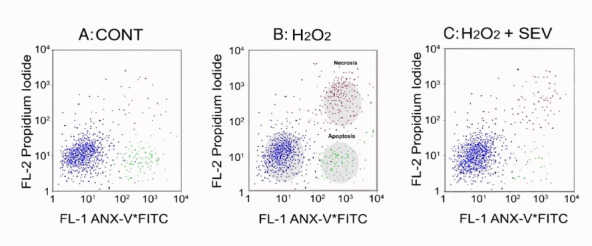
The flow-cytometry comparison of Annexin-fluorescein isothiocyanate (ANX-V + FITC) and Propidium iodide (PI). 2A: Controls; 2B: Reactive oxygen species (ROS); and 2C: ROS with Sevoflurane (SEV). The cells’ characteristics are as follows: normal cells blue (lower left quadrant); apoptotic cells: ANX-V + FITC positive cells (FL-1: green; lower right quadrant), necrotic cells: PI-positive (FL-2: Red, upper right quadrant)

**Figure 3:**
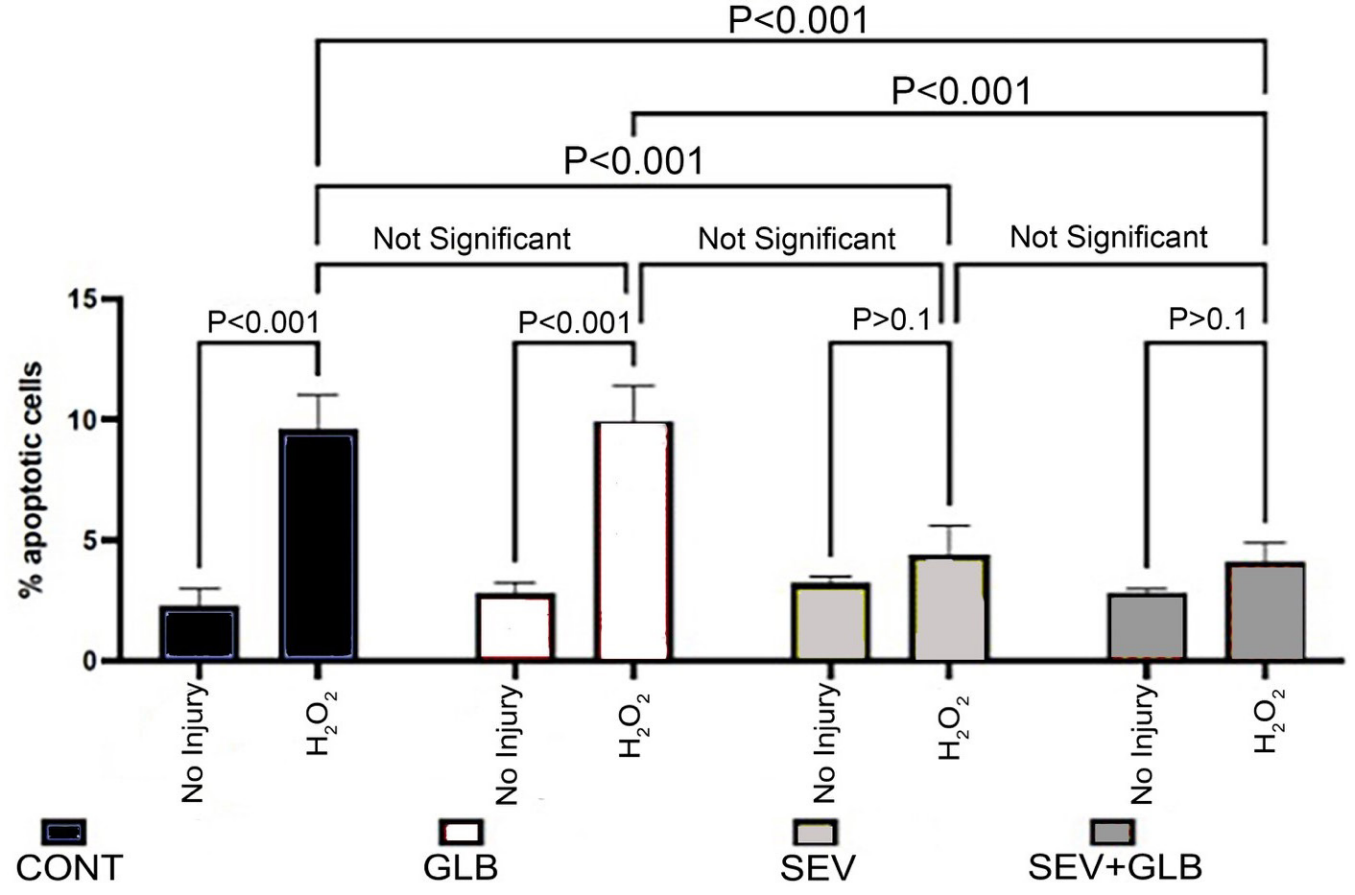
The percentage of apoptotic cells two hours after various treatments. CONT: Controls; H2O2: Reactive oxygen species; GLB: Glibenclamide and SEV: Sevoflurane

 Gross apoptotic activity: An increase in necrosis and apoptosis is appreciated when isolated left ventricular myocytes are exposed to oxidative injury (Figure 2B) compared to CONT (Figure 2A). Treating cells with SEV decreased the observed injury (Figure 2C). A closer look at apoptotic activity amongst all non-ROS exposed cells (Figure 3) demonstrates that a larger increase in apoptosis of the myocytes was observed following ischemia in the CONT and GLB treated groups (9.6 ± 1.4% and 9.9 ± 1.5%, respectively with *P* value < 0.001). The difference between the CONT group and the myocytes that received GLB was negligible (*P* value > 0.999). Under the same oxidative insult, treatment with SEV decreased the apoptotic rate in the myocytes appreciatively drastically (4.4 ± 1.2%) (*P* value < 0.001). Oxidative injury demonstrated a marginal increase in the apoptotic rate compared to its non-ROS-treated counterpart (*P* value = 0.613). Even when GLB was added to SEV under the same oxidative conditions, there was very minimal change in the apoptotic rate (4.1 ± 0.8%, *P* value > 0.999), and similar to SEV, only a marginal increase from its non-ROS treated counterpart (*P* value = 0.460). The degree of apoptosis was similar among all groups not exposed to ROS injury.

###  Caspase 3 Activity

**Figure 4:**
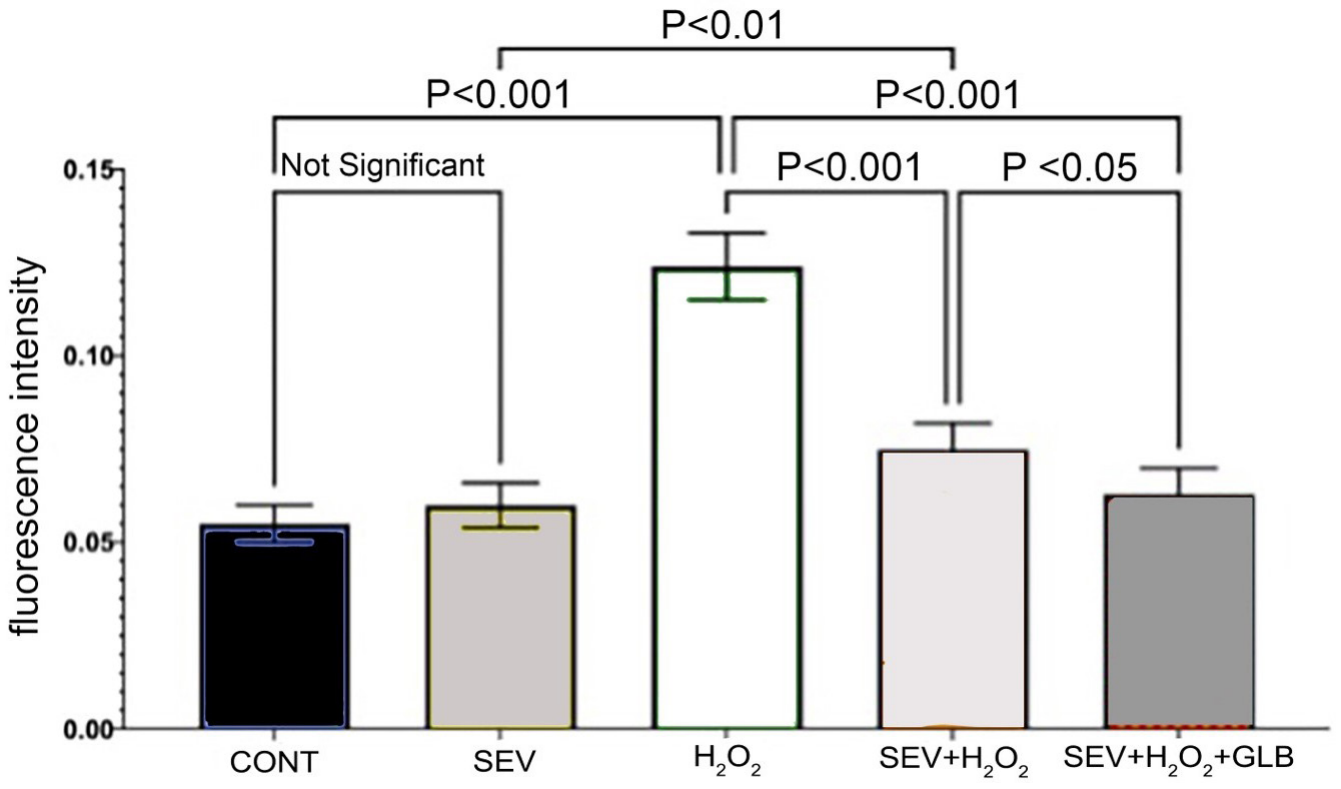
Caspase-3 activity two hours after various treatments. CONT: Controls; H2O2: Reactive oxygen species; GLB: Glibenclamide and SEV: Sevoflurane

 We also assessed increases in one of the chemical mediators of apoptosis, caspase 3 (Figure 4). The ROS-exposed myocytes in the CONT group had the highest mean caspase three fluorescence intensity (0.124 ± 0.009) compared to all groups under the same conditions (*P* value: < 0.001). This value decreased significantly when the ROS-exposed cells were treated with SEV (0.062 ± 0.006).

 Non-oxidant CONT (0.551 ± 0.005) and SEV-treated groups are comparable (*P* value = 0.758), serving as the CONTs. The no-treatment group had the highest level of caspase activity (0.124 ± 0.009). Treatment with SEV drastically reduced the mean caspase 3 activity (0.062 ± 0.006) (*P* value < 0.001). A similar effect was observed even in adding GLB to the SEV (0.063 ± 0.001; *P* value < 0.001). In oxidative injury, treatment with both GLB and SEV treatments further decreased caspase activity compared to SEV alone. Interestingly, comparing oxidative insult in myocytes exposed to both SEV + GLB was slightly lower than SEV under the same conditions (*p* value = 0.036). 

## Discussion

 We have demonstrated that treating isolated cardiomyocytes with sevoflurane diminishes apoptotic and necrotic cell damage secondary to an oxidative injury. ROS provoke apoptotic injury mainly through upregulating pro-inflammatory genes.^3,9-11^ We previously demonstrated that sevoflurane decreases inflammatory responses after cardiopulmonary bypass by showing a functional improvement in the heart post-reperfusion.^3^

 Reactive oxygen species cause necrotic and apoptotic cell damage through direct oxidizing effects on the macromolecules involved.^12^ This ROS-mediated damage underlies the pathogenesis of various diseases.^13,14^ For example, our laboratory demonstrated increased oxidant activity on lipids and proteins, causing lung injury following acid aspiration and high ambient oxygen concentrations.^12^ Periods of ischemia induce ROS in tissues triggering the activation of white blood cells (WBC). These activated WBCs can damage the involved tissues.^15^ Similarly, ROS-mediated apoptosis and necrosis of cardiomyocytes lead to heart failure following ischemia.^16,17^ In this study, we have shown that ROS leads to apoptosis and necrosis in isolated cardiomyocytes.

 Apoptosis is an actively regulated process of cellular destruction without inflammation. Although the associated ischemic insult is less than is required for necrosis, apoptosis may still confer considerable dysfunction, as is observed in the myocardium. In an ischemic heart, necrosis is more prominent within the first 24 hours.^16^ However, the apoptotic changes present late after the ischemic insult due to reperfusion. This degree of myocardial injury may be reversible (“stunning”) or irreversible, leading to uncompensated heart dysfunction.^16^ On a molecular level, caspase 3 (a primary proteolytic mediator of apoptosis) directly correlates with the extent of cell death through autophagic processes. Caspases are involved in apoptotic cell death via proteolysis within the cells.^6-8^

 K_ATP_ channels are strongly associated with apoptotic and necrotic cell injury. These channels open mainly during periods of energy depletion, as seen in ischemic events, thus mediating responses involved in stress adaptation.^18,19^ K_ATP_ channels are found on the cardiac myocytes sarcolemma and the mitochondrial membrane.^19,20^ Volatile anesthetics increase the opening of K_ATP_ channels leading to a decrease in the cytosolic and mitochondrial calcium load. This mechanism offers primary protection against calcium accumulation and mitochondrial dysfunction, as is seen with ischemia-reperfusion injury.^21,22^ Opening the K_ATP_ channels decreases the calcium influx and restores the mitochondrial membrane potential, thus preventing apoptosis. Opening further leads to an increased ATP synthesis which protects against cellular injury.^23,24^ The sarcolemmal K_ATP_ channels decrease the O_2_ and ATP consumption and may shorten the action potential, leading to myocardial protection.^23^

 Deactivating K_ATP_ channels with pharmacological inhibitors such as GLB hinders or diminishes ischemic and anesthetic preconditioning protection.^25-27^ Interestingly, unlike these studies with the deactivation of K_ATP_ channels, we demonstrated only minor attenuation of anesthetic preconditioning with SEV. Due to the complexity of anesthetic preconditioning, the causality of our contradicting results still needs to be fully understood. We hypothesize that K_ATP_ channels are not our model’s sole mediators associated with anesthetic protection.

 Mitochondrial permeability transition pores (MPTP) also play an essential role in apoptosis during a sustained injury. When these channels are in an irreversible open state, there is a disruption of the mitochondrial potential and subsequent swelling of the mitochondria. With the swelling of mitochondria, pro-apoptotic proteins such as cytochrome* c* are released into the cytosol triggering a cascade of events that lead to apoptosis or necrosis.^28^

 Similar to K_ATP_ channels, MPTPs are vital in myocardial preconditioning. Volatile anesthetics delay the opening of these channels under oxidative stress in rat cardiomyocytes and cardiomyocyte-derived embryonic stem cells, which leads to cellular protection.^29-33^ It is hypothesized that the incorporation of lipid-soluble volatile anesthetics reversibly into the lipid bilayer component of the cell membrane causes a lateral pressure favoring the closed state of the MPTPs, which may contribute to ischemia protection.^34^

 Additionally, volatile anesthetics may directly modulate anti-apoptotic genes. Preconditioning with volatile anesthetics for one hour provides neuroprotection by upregulating the anti-apoptotic genes.^35^ In rat neurons exposed to volatile anesthetics, an increased ratio of the anti-apoptotic Bcl-2 protein family to a decreased pro-apoptotic activation of the c-Jun N-terminal kinase (JNK) and p53 pathway led to a decrease in caspase-3 expression.^36^ The tandem of P-domains in weakly inward rectifying K^+^ channels (TWIK) is another set of ion channels associated with apoptotic cell death. TWIK-related acid-sensitive potassium channels (TASK) 1 through 3 inhibit the intracellular apoptotic pathways and thus enhance cellular viability. Volatile anesthetics augment these protective effects by enhancing the TASK3 activity.^37,38^

 Limitations in our study may include alterations induced by isolating cardiomyocytes on the pathways involved in apoptosis, such as that of K_ATP _channels. The isolation of the cells may alter many ways involved in physiological functions. Furthermore, the timing and dosage of the GLB may have been insufficient to deactivate the K_ATP_ channels in this model. Additionally, we have considered whether GLB should have been given post-isolation to ensure a compatible ratio of SEV and GLB in the system.

## Conclusion

 In a previous review article, we have outlined the immune modulatory effects of the volatiles anesthetics.^39^ Along with the many other studies conducted so far, only future studies and experimental work can answer the question of the different pathways associated with anesthetic preconditioning responsible for our findings. To investigate the direct effects of MPTPs on anesthetic preconditioning, the blockage of both K_ATP_ channels with the MPTPs may clarify the mechanism responsible for our results. Finally, to better understand the cellular mechanisms of volatile anesthetic action, hyperbaric pressure experiments may be promising as high atmospheric pressures are shown to reverse many volatile anesthetics actions.^40^

## Competing Interests

 None declared by the authors.

## Ethical Approval

 All experimental procedures and protocols used in this study were reviewed and approved by the Animal Use and Care Committee of the VA Medical Center at Buffalo (Approval letter No: 55443-16326 dated Feb 18, 2014), conforming to the Guiding Principles in the Care and Use of Animals of the American Physiologic Society.

## Funding

 Funding was provided for this study by the grant-in-aid from the American Heart Association (00604240) and the Society of Cardiothoracic Anesthesiology awarded to NDN.
